# Data on large cardamom transcriptome associated with Chirke disease

**DOI:** 10.1016/j.dib.2019.105047

**Published:** 2020-01-07

**Authors:** K. Mary Mathew, Ranjanan Reshma, M. Geethu, Varghese Rithin, Sasidharan Swapna, P.P. Gouthaman, K.K. Sabu, F. Nadiya, Muhammad Ali Noushad, Soumya S. Dharan, R. Prakashkumar, A.B. Remashree

**Affiliations:** a Indian Cardamom Research Institute, Spices Board Min Commerce & Industry, Government of India, Myladumpara, Idukki, Kerala 685553, India; b Jawaharlal Nehru Tropical Botanic Garden and Research Institute, Palode, Thiruvananthapuram 695562, India

**Keywords:** Large cardamom, RNA sequencing, Transcriptome, Differential expression

## Abstract

Large cardamom (*Amomum subulatum* Roxburg), is an ancient spice native to North-Eastern India and Southeast Asia, which belongs to the family *Zingiberaceae* under the order *Scitaminae.* Large cardamom is mostly affected by a viral disease termed Chirke caused by Large Cardamom Chirke Virus (LCCV). These disease has spread due to drastic changes in the ecosystem, inadequate rain in dry months and absence of good agricultural practices by the farmers resulting in aphid infestations. In the present study, using HiSeq™ 2000 RNA sequencing technology transcriptome sequencing was performed for both control (disease not expressed) and diseased large cardamom leaf tissues. RNA-seq generated 77260968 (7.72 GB) and 72239708 (7.22 GB) paired raw reads for large cardamom control and diseased samples respectively. The raw data were submitted to the NCBI SRA database under the accession numbers SRX2529373 and SRX2529372 and the assembled transcriptomes were submitted to TSA under the accession numbers GIAV01000000 and GIAW01000000 for the control and diseased samples respectively. The raw reads were quality trimmed and assembled *de novo* using TRINITY assembler which created 156822 (control) and 148953 (diseased) contigs with N50 values 2107 (control) and 2182 (diseased). The data were used to identify the significantly differentially expressed genes between control and diseased samples.

**Table:** Specifications Table

Subject	Agricultural and Biological Sciences
Specific subject area	Plant Science
Type of data	Text (FASTQ sequence files), table
How data were acquired	RNA sequencing data generated from Illumina HiSeq™ 2000
Data format	Raw data FASTQ format
Parameters for data collection	Freshly collected leaf samples from both control and naturally infected (diseased) large cardamom plants were used for RNA isolation.
Description of data collection	RNA seq libraries representing control and chirke disease stressed large cardamom were prepared, transcriptome sequencing was performed and *de novo* assembled to generate unigenes.
Data source location	Plants naturally infected at ICRI Regional Research Station, Gangtok in the East District of Sikkim, India (27° 18′ 41.724″ N, 88° 35′ 31.923″ E). Data was generated from Illumina HiSeq™ 2000
Data accessibility	Raw sequences of both control and disease stressed samples are available at NCBI SRA public repository: https://www.ncbi.nlm.nih.gov/sra/SRX2529373[accn] (control) https://www.ncbi.nlm.nih.gov/sra/SRX2529372[accn] (diseased) Transcriptome Shotgun Assembly for the control sample has been deposited at DDBJ/EMBL/GenBank under the accession GIAV00000000. The version described in this paper is the first version, GIAV01000000. Transcriptome Shotgun Assembly for the diseased sample has been deposited at DDBJ/EMBL/GenBank under the accession GIAW00000000. The version described in this paper is the first version, GIAW01000000.

**Table:** 

**Value of Data** • Large cardamom is severely affected by many diseases, prominent among them is chirke viral infection which affects the crop productivity. • Expression profiling could unravel the over-expression of R genes or genes related to plant stress tolerance. • Transcriptome data generated from leaves of plants grown under specific conditions could provide information on the molecular mechanism underlying disease tolerance. • Differential expression analysis of control and disease stressed large cardamom could compare the expression variation of particular genes in healthy and infected plants and can be utilized for several downstream applications.

## 1 Data

**Fig. 1:**
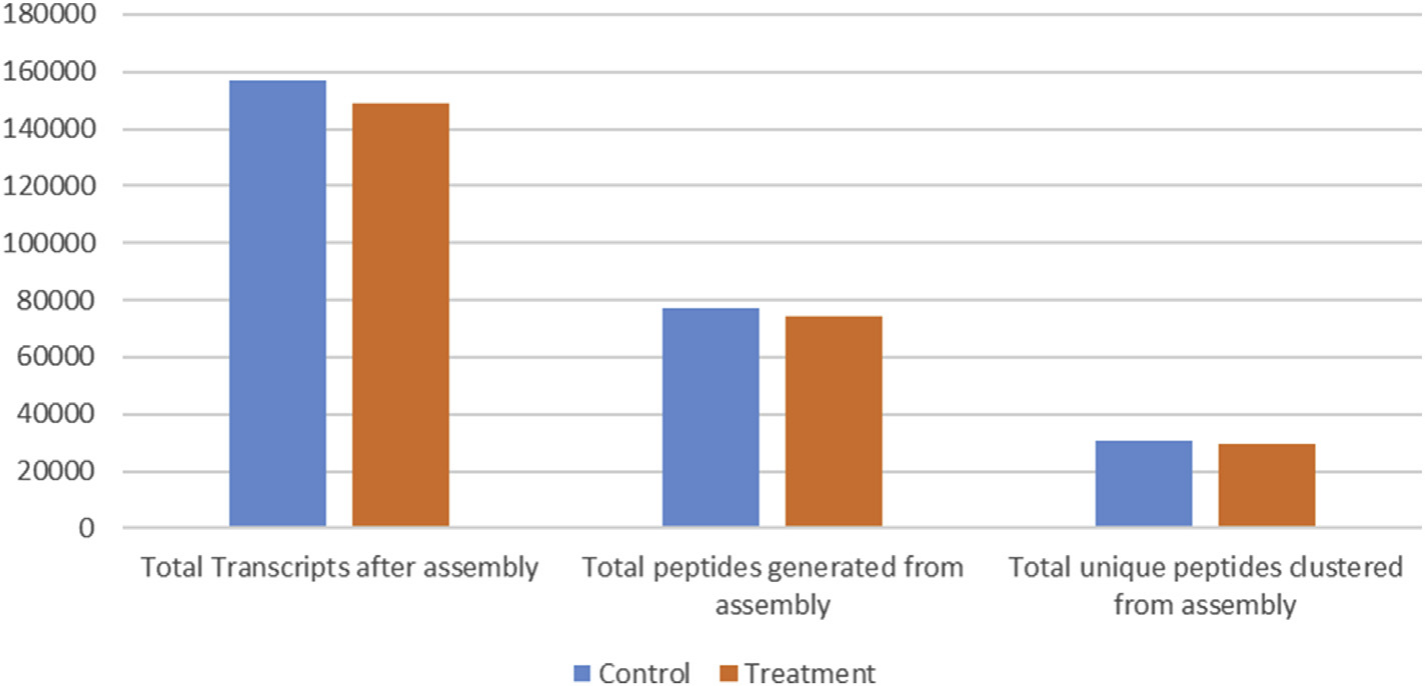
Representation of numerical difference in gene and peptide count among the control and treatment.

The dataset contains raw sequencing data obtained through transcriptome sequencing of leaf samples of large cardamom (*Amomum subulatum* Roxburg). The data files were deposited at NCBI SRA database under project accession no. PRJNA369131. Information generated from the raw data and that of assembly are provided in Table 1 and Fig. 1.

**Table 1:** Read and assembly statistics of control and infected large cardamom data.

Plant Material	Control	Diseased
Total number of raw reads	77260968	72239708
Total number of bases	7803357768	7296210508
Initial GC%	46	45
Read length	101	101
GC% after trimming	45.5	45
Reads after adapter removal and quality trimming	37733851	35199417
Total contigs	156822	148953
Largest contig	37547	23530
N50	2107	2182
L50	23639	22103
Total Length	172328012	167556334
GC% after assembly	41.97	42.11
Size of the assembly	168.3 MB	163.6 MB
Raw reads mapped to assembly (%)	97.70	97.17
Coverage	44.68	42.73
Scaffolds with any coverage (%)	98.83	99.00

## 2 Experimental design, materials, and methods

### 2.1 Plant material

Transcriptome sequencing was carried out in leaf samples of large cardamom (*Amomum subulatum* Roxburg). Large cardamom chirke virus (LCCV) was not expressed in one of the samples which served as the control whereas the disease was expressed in the other sample. Leaf tissues from both sets were collected followed by immediate freezing in liquid nitrogen.

### 2.2 Total RNA isolation and transcriptome sequencing

RNA extraction was done using a modified protocol of the RNeasy Plant Mini Kit (Qiagen) and CTAB method [1] RNA integrity and quality analysis were done using 2100 BioAnalyzer (Agilent Technologies). Illumina sequencing was performed using the HiSeq™ 2000 platform as per the manufacturer's instructions (Illumina, San Diego, CA). RNA-seq generated paired-end strand-specific 77260968 (101 bases) and 72239708 (101 bases) raw reads which correspond to 7.72 GB and 7.22 GB of sequence data for large cardamom control and diseased samples respectively.

### 2.3 *De novo* transcriptome assembly

Raw reads were first quality checked using the FastQC [2] tool and the different criteria were cross-checked to determine the integrity of the raw data and based on the quality control data it was determined to trim the raw reads of any adapters present in it. Adapter trimming was done using BBDuk [3] against Illumina universal adapters. Non-coding RNAs such as tRNAs, rRNAs, snRNAs, and snoRNAs were filtred using BBSplit [3] against all non-coding RNA sequences of viridiplantae collected from NCBI, based on further quality checking it was determined that the data was ready for assembly. *De novo* transcriptome assembly was performed using the Trinity [4] assembler program (Trinity Release v 2.8.5) utilizing three consecutive modules: Inchworm, Chrysalis, and Butterfly to generate contigs. The assembler created 156822 and 148953 contigs for control and infected large cardamom samples (Table 1). The assembled transcripts were converted into peptides using Transdecoder [5] and the peptides were clustered using cd-hit [6] to produce non-redundant and representative sequences. Further statistical data were generated from the assembly by means of the QUAST tool [7].

### 2.4 Confirmation of chirke virus genome sequences in the assembled transcriptome

Virus genome sequences were fetched from NCBI (https://www.ncbi.nlm.nih.gov/nuccore/?term=chirke) and found only 4 sequences for chirke (JN257715.1, MH899149.1, MH899148.1, and MH899147.1). These were aligned to both infected and control sequences using BLAST+ [8]. The Alignment generated 140 hits for the infected sequences. Whereas the control sequence showed one hit from all four of the sequences. This might be due to the dormant virus particles present in the control sequences or possible cross-contamination.

### 2.5 Quantification of peptides from the transcripts

A total of 156822 transcripts were generated from the control sample while 148953 were generated from the diseased. While converting the transcripts into peptides the control sample generated 76913 peptide sequences while the treatment generated 74060. The obtained peptides were clustered for non-redundancy which resulted in 30498 unique peptides being generated from control compared to the 29512 that were generated from the diseased (Fig. 1).
